# Integrated machine learning developed a prognosis‐related gene signature to predict prognosis in oesophageal squamous cell carcinoma

**DOI:** 10.1111/jcmm.70171

**Published:** 2024-11-13

**Authors:** Peng Tang, Baihui Li, Zijing Zhou, Haitong Wang, Mingquan Ma, Lei Gong, Yufeng Qiao, Peng Ren, Hongdian Zhang

**Affiliations:** ^1^ Department of Esophageal Cancer Tianjin Medical University Cancer Institute & Hospital, National Clinical Research Center for Cancer, Tianjin's Clinical Research Center for Cancer, Tianjin Key Laboratory of Digestive Cancer Tianjin China; ^2^ Department of Radiation Oncology Tianjin Medical University Cancer Institute & Hospital, National Clinical Research Center for Cancer, Tianjin's Clinical Research Center for Cancer, Key Laboratory of Cancer Prevention and Therapy Tianjin China

**Keywords:** machine‐learning algorithm, oesophageal squamous cell carcinoma, predictive model, random survival forest, tumour‐infiltrating immune cells

## Abstract

The mortality rate of oesophageal squamous cell carcinoma (ESCC) remains high, and conventional TNM systems cannot accurately predict its prognosis, thus necessitating a predictive model. In this study, a 17‐gene prognosis‐related gene signature (PRS) predictive model was constructed using the random survival forest algorithm as the optimal algorithm among 99 machine‐learning algorithm combinations based on data from 260 patients obtained from TCGA and GEO. The PRS model consistently outperformed other clinicopathological features and previously published signatures with superior prognostic accuracy, as evidenced by the receiver operating characteristic curve, C‐index and decision curve analysis in both training and validation cohorts. In the Cox regression analysis, PRS score was an independent adverse prognostic factor. The 17 genes of PRS were predominantly expressed in malignant cells by single‐cell RNA‐seq analysis via the TISCH2 database. They were involved in immunological and metabolic pathways according to GSEA and GSVA. The high‐risk group exhibited increased immune cell infiltration based on seven immunological algorithms, accompanied by a complex immune function status and elevated immune factor expression. Overall, the PRS model can serve as an excellent tool for overall survival prediction in ESCC and may facilitate individualized treatment strategies and predction of immunotherapy for patients with ESCC.

## 1 INTRODUCTION

Oesophageal squamous cell carcinoma (ESCC), a common type of oesophageal cancer globally, is characterized by its aggressive local invasion, lymph node metastasis at diagnosis and high mortality rates.^1^, ^2^, ^3^ Predictive models have been developed in ESCC, utilizing multiple factors to predict recurrence,^4^ lymph node metastasis,^5^ and outcomes.^4^, ^5^, ^6^, ^7^, ^8^, ^9^, ^10^, ^11^, ^12^, ^13^ Many indicators, including clinical parameters,^4^, ^5^, ^8^, ^9^, ^10^, ^12^, ^13^ tumour pathological characteristics,^4^, ^5^, ^8^, ^9^, ^10^, ^12^, ^13^ imaging data,^6^, ^9^ and haematological indices,^11^ can be incorporated into constructing prognostic models. The rapid advancement of next‐generation sequencing has recently improved the stratified management and accurate treatment of ESCC.^3^, ^14^, ^15^, ^16^ In addition to incorporating traditional variables, other models were constructed to predict clinical outcomes by integrating genomic,^14^ epigenomic,^15^, ^16^ transcriptomic,^7^, ^17^, ^18^, ^19^, ^20^, ^21^, ^22^, ^23^, ^24^ and multi‐omics data.^3^ These models could provide personalized treatment strategies, survival predictions, optimized follow‐up strategies and clinical decision support and even promote research and innovation in cancer treatment.^8^, ^9^, ^10^ However, predictive models for ESCC have not been fully developed and still exhibit many shortcomings, possibly due to the disease heterogeneity and variability of individual clinical courses. Therefore, there remains an urgent need for predictive models to improve survival outcomes in patients with ESCC.

Machine learning (ML), which is a subset of artificial intelligence, shows significant promise in oncology for early detection, disease classification and personalized treatment approaches.^25^, ^26^ It also is a compelling approach with the potential to transform the manner in which cancer prognosis is assessed and predicted.^27^, ^28^ ML algorithms, includings random survival forest (RSF), decision tree, survival support vector machine (Survival‐SVM), or deep learning methods, can be utilized to develop more precise predictive models.^25^, ^26^, ^27^, ^28^ However, only a few studies on ESCC have adopted ML methods.^4^, ^5^, ^11^, ^12^, ^13^ Furthermore, these studies included only 3–6 types of ML algorithms, the involved model variables were limited in clinical parameters or pathological characteristics and the patient sample size was not adequate. Currently, an increasing number of ML algorithm combinations are used to develop predictive models in cancer.^27^, ^28^, ^29^ Hence, predicting clinical outcomes and identifying optimal treatments for patients with ESCC remain significant challenges and fully utilizing ML methods to construct a comprehensive predictive risk model in a large sample cohort is highly important. This study aimed to develop a model to predict the prognosis and guide clinical treatment decisions for ESCC via integrating 10 classical algorithms and 99 ML combinations.

**FIGURE 1:**
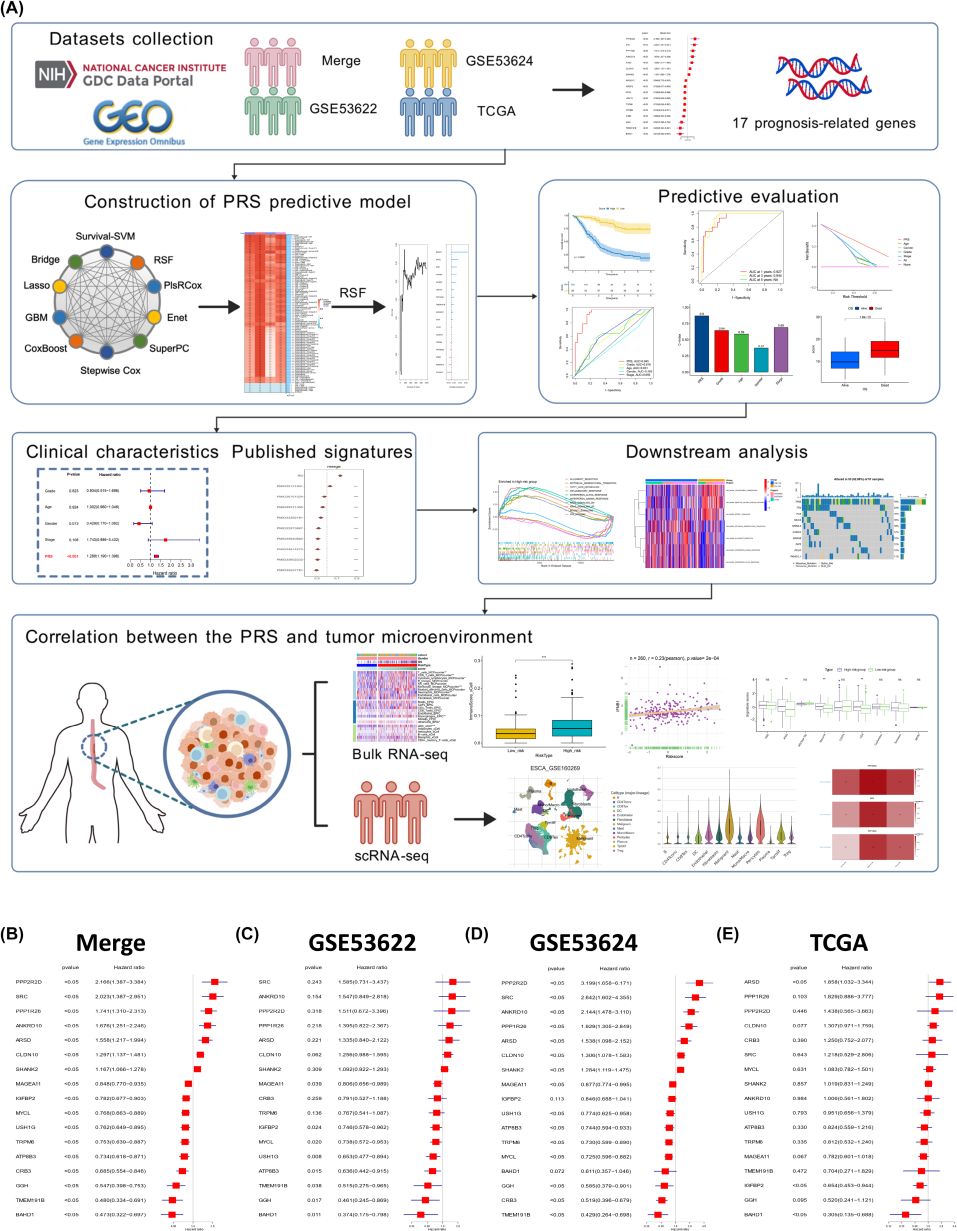
Screening for prognostic genes in ESCC using univariate Cox analysis. (A) A workflow of this study illustrated the overall process across the study, which created with BioGDP.com. (B) Univariate Cox regression analysis identified that 7 genes were associated with poor prognosis (*p* < 0.001, HR >1) and that 10 genes were associated with good prognosis (*p* < 0.001, HR <1) in the Merge dataset. (C) These genes were further verified in the GSE53622, (D) GSE53624 and (E) TCGA cohorts.

In this study, a novel predictive model for overall survival (OS) in patients with ESCC was constructed using the RSF algorithm. The overall study process is illustrated in Figure 1A. The prognosis‐related signature (PRS) predictive model proved an independent and robust prognostic factor that outperformed the conventional TNM staging system and previous predictive models in available ESCC datasets. This study proposes the PRS predictive model as a powerful tool for OS prediction and will provide strong support for personalized treatment and immunotherapy prediction in patients with ESCC.

## 2 MATERIALS AND METHODS

### 2.1 ESCC sample data collection

The RNA‐seq datasets comprised TCGA ESCC, GSE53622 and GSE53624.FPKM‐normalized RNA‐seq expression data and clinical information from TCGA were downloaded from the UCSC Xena website (https://xenabrowser.net/). GEO data were downloaded from GEO database (https://www.ncbi.nlm.nih.gov/geo/). A total of 260 samples were enrolled and the follow‐up time was more than >0 days; 81 samples were obtained from TCGA, 60 from the GSE53622 cohort and 119 from the GSE53624 cohort. To combine the different datasets, the ‘removeBatchEffect’ function in the ‘limma’ package and the ‘Combat’ function in the ‘SVA’ package was employed to adjust the bath effect in each dataset.^30^, ^31^ A principal‐component analysis (PCA) was conducted to assess the efficacy of the correction prior to and following the removal of batch effects.^32^ All data were log‐transformed prior to analysis. Somatic mutation and copy number variation (CNV) data for patients with TCGA‐ESCC were obtained from the UCSC Xena website.

### 2.2 Identification of PRS by ML

First, univariate Cox regression analysis was applied to identify PRS, namely 17 genes with potential prognostic significance in the combined dataset (| hazard ratio (HR) > 1|, *p* < 0.001). TCGA ESCC, GSE53622 and GSE53624 cohorts were used as validation sets. To construct a predictive model with high generalizability and accuracy, 10 classical algorithms were integrated: RSF, least absolute shrinkage and selection operator (LASSO), Survival‐SVM, gradient boosting machine (GBM), supervised principal component (SuperPC), CoxBoost, Ridge regression, partial least squares regression for Cox (plsRcox), stepwise Cox and elastic network (Enet) algorithms.^28^, ^33^ Ninety‐nine ML combinations of 10 algorithms were arranged in the training dataset GSE53622 to screen variables and construct a prognostic predictive model. A ten‐fold cross‐validation framework was carried out to fit 99 prediction models, with the concordance index (C‐index) used to evaluate the model's predictive accuracy using the ‘survcomp’ package in the GSE53622. Finally, all constructed models were assessed using TCGA ESCC, GSE53624 and a combined dataset. The C‐index was calculated for the predictive performance across training and validation datasets to identify the most superior algorithm combination.^28^ Ultimately, the RSF was defined as the optimal algorithm with the highest average C‐index. The PRS was established to predict the OS of patients with ESCC. More detailed methodological steps are available in the supplementary file.

### 2.3 Assessment of the PRS prediction accuracy

In both the training and validation datasetspatients were divided into high‐ or low‐risk groups, according to the optimal cut‐off of the PRS risk score in GSE53622 dataset using the ‘maxstat’ R package. Then, Kaplan–Meier (KM) survival analysis was conducted by the R packages ‘survival’ and ‘surviminer’ to evaluate the prognostic significance of the PRS for OS with the log‐rank test (*p* < 0.05). Moreover, receiver operating characteristic (ROC) curves, incorporating clinical characteristics, were constructed to evaluate the sensitivity and specificity of the PRS prognostic efficacy using the ‘timeROC’ package and the area under the curve (AUC) was calculated by the ‘survivalROC’ package. Time‐dependent AUC was computed to represent the average AUC over time. The C‐index was estimated to compare the predictive performance of PRS and previously published modles. Decision curve analysis (DCA) was conducted to estimate the clinical benefit to patients by ‘ggDCA’ package. Univariate and multivariate Cox regression analyses were performed to explore the independent prognostic factor via ‘survival’ package.

### 2.4 Biological function and pathway enrichment analysis

To elucidate the underlying mechanisms of PRS, Gene Ontology (GO) and Kyoto Encyclopedia of Genes and Genomes (KEGG) analyses were performed by the ‘clusterProfiler’ R package by genes associated with the PRS (*p* < 0.05, Top 50). Gene set enrichment analyse (GSEA) was conducted by the ‘clusterProfiler’ and ‘enrichplot’ R packages with GSEA 4.3.2 (http://www.broadinstitute.org/gsea/). Gene set variation analysis (GSVA) was performed by the ‘GSVA’ R package.^34^ The Go enrichment analysis results were reported for thebiological process (BP), cellular component (CC) and molecular function (MF).

### 2.5 Comprehensive analysis of immune microenvironment features

To assess the extent of immunological cell infiltration, seven bioinformatic algorithms were utilized, namely, Microenvironment Cell Populations‐counter (MCPcounter), xCell, Estimation of Proportion of Immune and Cancer cells (EPIC), Cell‐type Identification by Estimating Relative Subpopulations of RNA Transcripts (CIBERSORT), Estimation of Stromal and Immune cells in Malignant Tumour tissues using Expression Data (ESTIMATE), QUANTISEQ and Tumour Immune Estimation Resource (TIMER) with the ‘IOBR’ R package.^35^ The significant differences in these immune‐related factors were visualized as a heatmap via the ‘Complex Heatmap’ R package. TIDE, IFN‐g (IFNG), microsatellite instability (MSI), Merck18 (T‐cell‐inflamed signature), CD8, CD274, Exclusion, Dysfunction, MDSC, CAF and TAM M2 scores were collected from the TIDE website (http://tide.dfci.harvard.edu).^36^ The single‐cell analysis of PRS was analysed on the Tumour Immune Single‐cell Hub (TISCH) website 2 (http://tisch.comp‐genomics.org/home/), a single‐cell RNA‐seq (scRNA‐seq) database that focuses on the characteristic of tumour immune microenvironment (TIME).^37^

### 2.6 Prediction of the PRS for drug sensitivity

The ‘oncoPredict’ R package was utilized to assess the half‐maximal inhibitory concentration (IC50) values of multiple anticancer drugs and the IC50 values were compared between high‐ and low‐risk groups. A higher IC50 indicates lower sensitivity to the treatment.

### 2.7 Statistical analysis

Statistical analyses and graphical illustrations were performed by R software (version 4.3.1) and R Studio (version 4.2.3). Wilcoxon test was conducted to compare nonnormally distributed variables between high‐ and low‐risk groups. The chi‐square test or Fisher's exact test was employed to valuate the correlation between the PRS score and clinical characteristics. Pearson correlation analysis was used to investigate the relationships between the PRS scores and various variables. A *p‐*value less than 0.05 (*), less than 0.01 (**) and less than 0.001 (***) were considered statistically significant.

## 3 RESULTS

### 3.1 Screening for prognostic genes in ESCC using univariate Cox analysis

The PCA plots demonstrated the successful removal of batch effects before and after the data merging (Figure S1A,S1B). The datasets were combined into a unified dataset called the Merge dataset. Subsequently, univariate Cox regression analysis was employed to identify the genes associated with prognosis (Figure 1B). Seven genes were associated with poor prognosis (*p* < 0.001, HR >1) and 10 with good prognosis (*p* < 0.001, HR <1) in the Merge dataset (Figure 1B). These genes were further verified in the GSE53622 (Figure 1C), GSE53624 (Figure 1D) and TCGA (Figure 1E) datasets. GO term annotation and KEGG pathway analyses were also conducted for these 17 genes (Figure S1C–S1F), revealing their primary involvement in cell junctions.

### 3.2 Machine learning for construction and validation of the PRS predictive model

**FIGURE 2:**
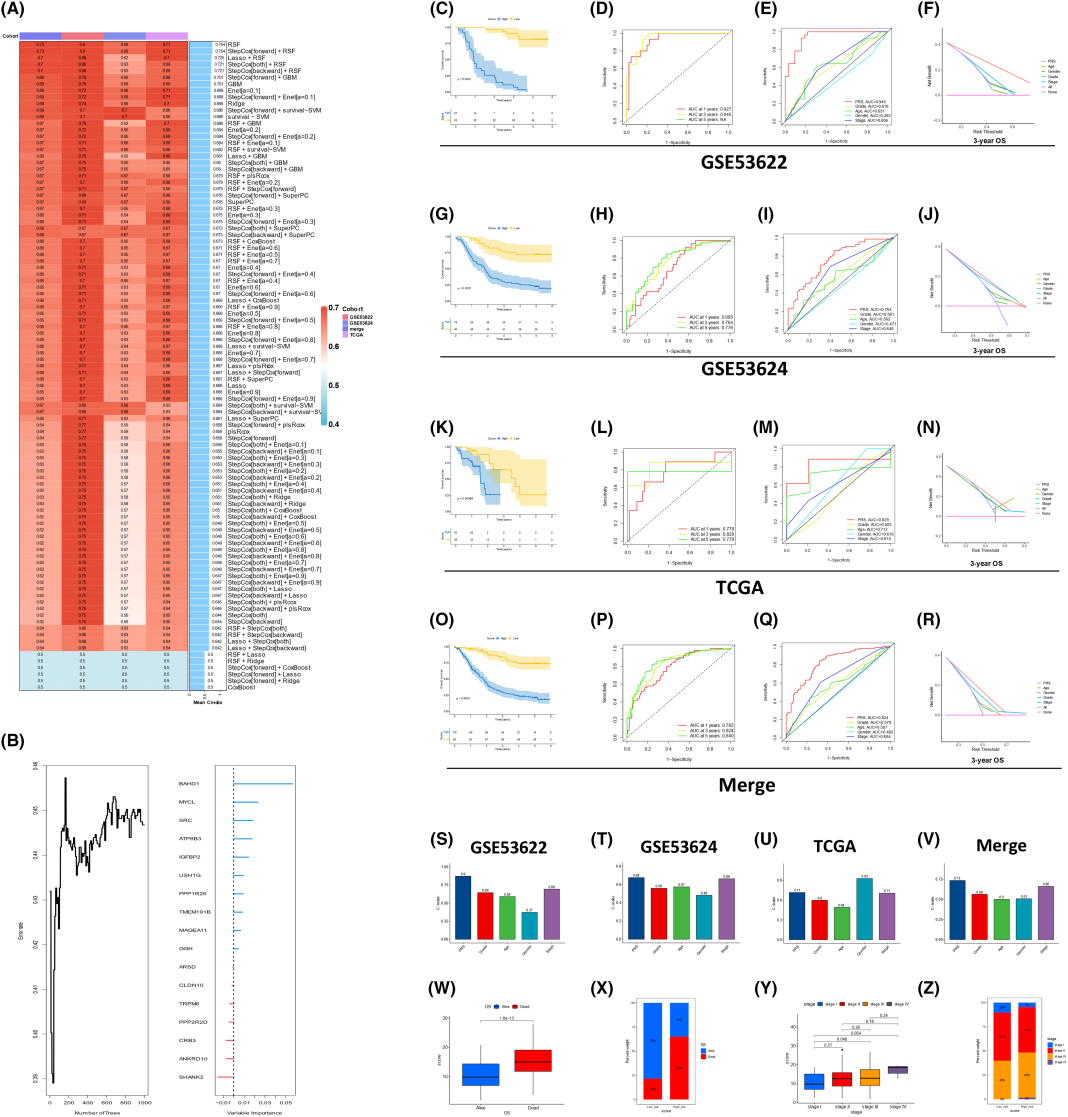
ML for constructing and validating of the prognosis‐related genes. (A) After integrating 10 classical algorithms and 99 machine learning combinations, RSF which had the highest average C‐index. (B) Genes identified according to the importance of the variables and the construction of an RSF algorithms model. (C) In the GSE53622 dataset, patients with high‐risk scores had significantly shorter OS than those with low‐risk scores. (D) The ROC‐AUC valuesfor predicting 1‐ and 3‐year OS. (E) The predictive efficacy of clinical parameters for predicting 3‐year OS. (F) DCA diagram for predicting 3‐year survival. (G) Patients with high‐risk scores had a notaly shorter OS than those with low‐risk scores in the GSE53624, (K) TCGA and (O) Merge cohorts. (H) The AUCs predicting 1‐, 3‐ and 5‐year OS in the GSE53624, (L) TCGA and (P) Merge datasets. (I) The predictive performance of clinical parameters for 3‐year OS across the GSE53624, (M) TCGA and (Q) Merge datasets. (J) The DCA plot for the 3‐year OS prediction of the PRS model in the GSE53624, (N) TCGA and (R) Merge datasets. The C‐index of the PRS model in the (S) GSE53622, (T) GSE53624, (U) TCGA and (V) Merge datasets. (W, X) The PRS scores were higher for ESCC patients who died than for those who were alive. (Y, Z) Patients with stage III disease exhibited significantly higher PRS scores than those with stage I disease.

These 17 genes were subsequently subjected to ML‐based integration to develop a PRS predictive model. To overcome the limitations of algorithm selection, 10 classical algorithms and 99 ML combinations were integrated to construct a predictive model in the GSE53622 training cohort and C‐index of every algorithm was further calculated across all the datasets (Figure 2A). The RSF algorithm was selected as the best method with the highest C‐index (0.754). A multivariable risk predictive model were constructed by 17 genes using RSF for OS in the GSE53622 cohort and their corresponding weights were shown on the right (Figure 2B). According to the GSE53622 dataset, patients with high risk scores had a significantly shorter OS than those with low‐risk scores (Figure 2C). ROC analysis displayed excellent accuracy, with the AUC values of 0.927 and 0.945 for predicting 1‐ and 3‐year OS, respectively (Figure 2D). According to the multivariate ROC curve analysis of 3‐year survival, the predictive efficacy of clinical parameters, such as grade, age, gender and stage, was notably lower than that of the PRS model (Figure 2E). A DCA diagram for predicting the 3‐year survival further demonstrated that the PRS model had a better evaluative impact than the other indicators (Figure 2F).

Consistent prognostic implications were observed in the validation datasets (Figure 2G–Z). Patients with high risk scores exhibited significantly shorter OS compared to those with the low risk scores in the GSE53624 (Figure 2G), TCGA (Figure 2K) and Merge datasets (Figure 2O). The AUCs for predicting 1‐, 3‐ and 5‐year OS were 0.695, 0.764 and 0.776 in the GSE53624 dataset (Figure 2H); 0.778, 0.829 and 0.779 in the TCGA dataset (Figure 2L); and 0.782, 0.824 and 0.840 in the Merge dataset (Figure 2P). The multivariate ROC curves for the prediction of 3‐year survival indicated that the predictive performance of the clinical parameters was significantly inferior to that of the PRS model in GSE53624 (Figure 2I), TCGA (Figure 2M) and Merge datasets (Figure 2Q). The results also showed that PRS was an effective predictive model with excellent sensitivity and specificity in both the training and validation cohorts. Moreover, the DCA plot for 3‐year survival prediction further revealed the superior evaluative impact of the PRS model compared with the alternative parameters in the GSE53624 (Figure 2J), TCGA (Figure 2N) and Merge datasets (Figure 2R). Moreover, the C‐index of PRS model was significantly higher than that of clinical parameters, including tumour grade, age, gender and stage, across GSE53622 (Figure 2S), GSE53624 (Figure 2T), TCGA (Figure 2U) and Merge datasets (Figure 2V). A notable exception was observed in TCGA dataset, where gender exhibited the highest C‐index.

The relationship between PRS and clinical parameters was further explored in the Merge dataset. Stratified survival analysis further validated the predictive role of the PRS model within different ESCC subgroups (Figure S2A–H). Additionally, the PRS scores were higher in patients who died than those who survived (Figure 2W,X). Notably, patients with stage III exhibited significantly higher PRS scores in contrast to those with stage I (Figure 2Y,Z). However, no statistical difference was observed among different cohorts (Figure S2I, J) or between genders (Figure S2K, L).

In summary, the 17‐gene PRS predictive model derived from the RSF algorithm consistently outperformed the TNM staging system and other clinical parameters in predicting OS across diverse ESCC datasets. The superior C‐index and ROC curve highlighted the potential of the PRS model as a highly effective predictive tool for patients with ESCC.

### 3.3 Cox regression analyses of the PRS score and clinical parameters

**FIGURE 3:**
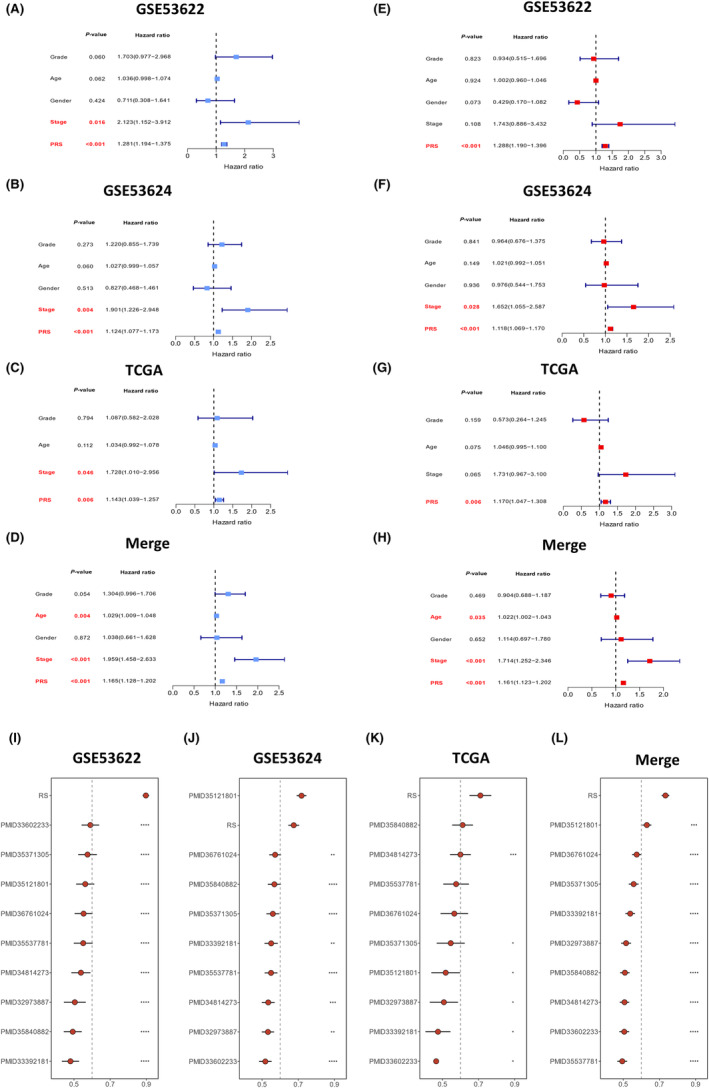
Cox regression for the PRS and comparisons with prior ESCC signatures. Based on univariate Cox analysis, PRS and stage were related to poor prognosis across (A) GSE53622, (B) GSE53624 and (C) TCGA datasets. (D) PRS, stage and age were associated with poor prognosis in the Merge dataset. Multivariate Cox analysis of the (E) GSE53622, (F) GES53624, (G) TCGA and (H) Merge datasets demonstrated that PRS independently served as a prognostic factor in ESCC patients. (I) The C‐index of the PRS predictive model ranked first among the other previously published signatures in the GSE53622, (K) TCGA and (L) Merge datasets. (J) The C‐index of the PRS model ranked second in the GSE53624 cohort, with no significant difference from that of the first‐ranked model.

Furthermore, univariate (Figure 3A–D) and multivariate (Figure 3E–H) Cox regression analyses were performed to identify whether the predictive efficacy of the PRS score was independentprognostic factors. Univariate Cox analysis revealed that the PRS score and stage correlated with poor prognosis in the GSE53622, GSE53624 and TCGA datasets (Figure 3A–C). The PRS score, stage and age correlated with poor prognosis in the Merge dataset (Figure 3D). Multivariate Cox analysis identified the PRS score as in independent prognostic factor across all datasets (Figure 3E–H), while stage emerged as an independent factor of prognosis in GSE53624 and Merge datasets (Figure 3F,H) and age independently influenced prognosis in the Merge dataset (Figure 3H). Although stage emerged as a prognostic factor analysed by the univariate Cox regression test in the training and validation cohorts (Figure 3A–D), its significance was observed only in the two validation cohorts analysed by the multivariate Cox regression test (Figure 3F,H). In contrast, univariate and multivariate Cox regression analyses demonstrated the predictive significance of the PRS score for OS in both the training and validation cohorts (Figure 3A–H), thus highlighting the stability of the PRS model.

In conclusion, the PRS score served as an independent prognostic factor with stronger implications than the traditional stage classification and other clinical parameters.

### 3.4 Comparisons between PRS and prior published signatures in patients with ESCC

Several studies have developed various gene signatures to predict the prognosis of ESCC.^6^, ^7^, ^9^, ^10^, ^12^, ^13^, ^17^, ^18^, ^19^, ^20^, ^21^, ^22^, ^23^, ^24^ To assess the predictive performance of the PRS in comparison to other gene signatures, a comprehensive literature search was performed to collect seven previously published gene signatures in ESCC,^17^, ^18^, ^19^, ^20^, ^21^, ^22^, ^23^ and two gene signatures from other cancers.^38^, ^39^ The predictive abilities of the PRS and these nine features were subsequently compared in both training and validation cohorts (Figure 3I–L). The C‐index results revealed that a few signatures performed well in only specific datasets, such as those from PMID33602233 in GSE53622, PMID35121801 in GSE53624 and PMID35840882 in the Merge dataset, but exhibited poor performance in other datasets. However, the PRS model showed significantly superior accuracy compared with almost all models across all datasets, confirming the stability and high accuracy of the PRS model. The PRS model achieved the highest rank in three datasets (Figure 3I,K,L) and second rank in one dataset with no significant difference from the first‐ranked model (Figure 3J).

In summary, the 17‐gene PRS predictive model exhibited superior accuracy and predictive performance across all datasets, surpassing prior predictive models and affirming its stability and precision with the highest top rankings.

### 3.5 Potential biological functions and pathways related to the PRS predictive model

To determine the underlying mechanisms of the 17‐gene PRS predictive model, comprehensive analyses were conducted in the Merge dataset (Figures S3 and 4). Heatmaps were used to visually represent the top 50 genes with positive correlation (Figure S3A) and negative correlations (Figure S3B). GO enrichment analysis showed that genes positively associated with the PRS (top 500 genes) were predominantly involved in cilium functions and antigen processing and presentation in terms of BP (Figure S3C). Among the CC terms, ‘membrane’ was the primary representation (Figure S3D), while among the MF terms, peptide antigen binding and MHC protein complex binding was the most common (Figure S3E). KEGG analysis results demonstrated that these genes were primarily related to antigen processing and presentation, infection and cell adhesion molecules (CAMs) (Figure S3F).

**FIGURE 4:**
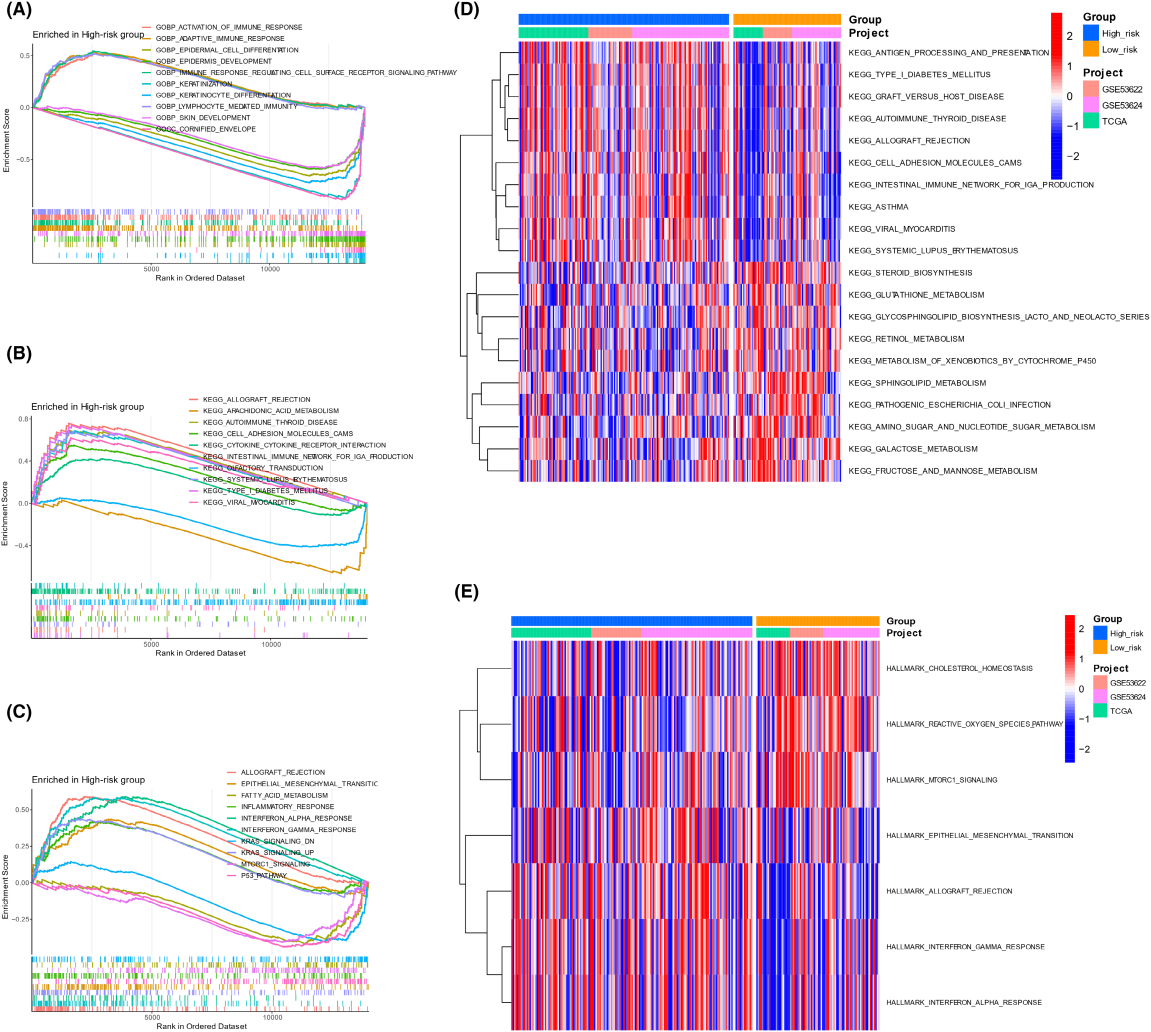
Potential biological functions and pathways associated with the PRS according to GSEA and GSVA. (A) The adaptive immune response, activation of the immune response, immune response regulating the cell surface receptor signalling pathway, and lymphocyte mediated immunity were enriched in the high‐risk group based on GSEA‐GO. (B) CAMs, cytokine and cytokine receptor interactions, the intestinal immune network for IgA production and immune‐related diseases were enriched in the high‐risk group according to GSEA‐KEGG. (C) The IFN‐r response, IFN‐a response, KARS signalling up, epithelial mesenchymal transition and inflammatory response were enriched in the high‐risk group according to GSEA‐Hallmark. (D) Compared with those in the low‐risk group, the GSVA scores for immune‐related pathways were elevated in the high‐risk group (E) Similar results were observed in the Hallmark pathway enrichment analysis.

Additional GSEA and GSVA were conducted to confirm these results (Figure 4). The GSEA‐GO results revealed enrichment of the immune response regulating cell surface receptor signalling pathway, adaptive immune response, activation of the immune response and lymphocyte mediated immunity in the high‐risk group. However, the low‐risk group exhibited enrichment of keratinization, cornified envelope, keratinocytes, epidermal cells, epidermis and skin differentiation (Figure 4A). The GSEA‐KEGG results indicated the enrichment of CAMs, intestinal immune network for IgA production, cytokine and cytokine receptor interaction and immune‐related diseases in high‐risk group. In contrast, arachidonic acid metabolism was enriched in low‐risk group (Figure 4B). GSEA‐Hallmark results demonstrated that the IFN‐ a response, IFN‐r response, KARS signalling up, epithelial mesenchymal transition and inflammatory response were increased in the high‐risk group. In contrast, the low‐risk group exhibited enrichment of the P53 pathway, mTORC1 signalling, fatty acid metabolism and KRAS signalling (Figure 4C).

Furthermore, the high‐risk group showed significantly elevated GSVA scores in immune‐related pathways, specifically antigen processing and presentation and intestinal immune network for IgA production, in comparison to the low‐risk group. Conversely, the low‐risk group exhibited significantly higher GSVA scores than the high‐risk group for metabolism‐related pathways, including steroid biosynthesis, glutathione metabolism, metabolism of xenobiotics by cytochrome P450, glycosphingolipid biosynthesis, amino sugar and nucleotide sugar metabolism, sphingolipid metabolism, galactose metabolism, retinol metabolism and fructose and mannose metabolism (Figure 4D). Consistent findings were observed in the Hallmark pathway enrichment analysis (Figure 4E).

Taken together, the high‐risk group exhibited enrichment in immune‐related pathways, notably the adaptive immune response and antigen processing and presentation pathway. In contrast, the low‐risk group showed enrichment in metabolism‐related pathways, such as those involved in steroid biosynthesis and glycosphingolipid biosynthesis.

### 3.6 Immune microenvironment and characteristics in different PRS score risk subgroups

**FIGURE 5:**
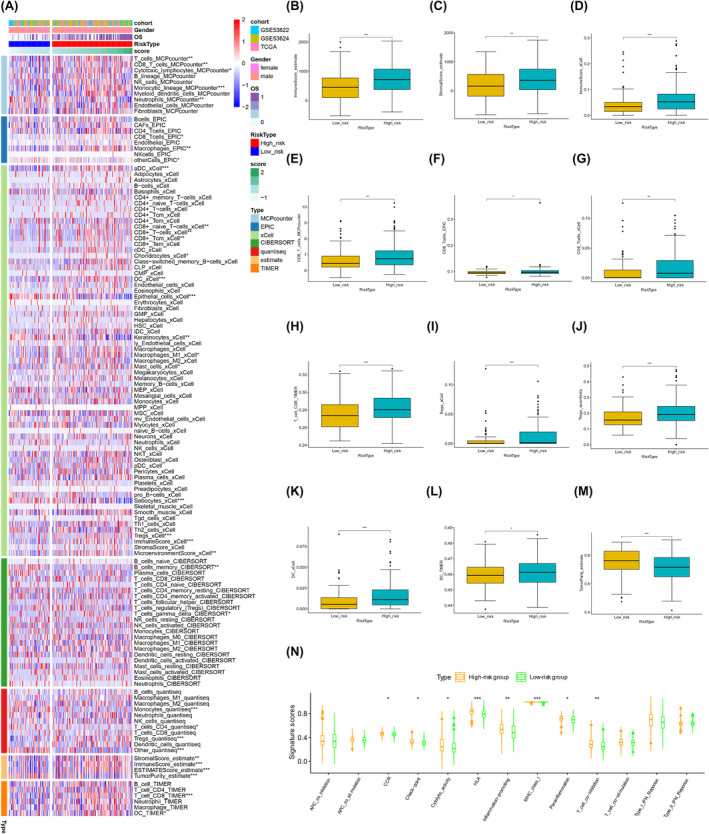
Immune microenvironment and characteristics in different PRS subgroups. (A) Seven immune algorithms were used to estimate the abundance of TIICs comprehensively. (B) The high‐risk group exhibited a significantly elevated ImmuneScore and (C) StromalScore according to the ESTIMATE algorithm. (D) The xCell algorithm revealed a higher ImmuneScore in the high‐risk group. (E) CD8^+^ T cells exhibited greater infiltration in the high‐risk group, as indicated by the MCPcounter, (F) EPIC, (G) xCell and (H) TIMER algorithms. (I) Tregs displayed increased infiltration in the high‐risk group, which was particularly evident in the xCell and (J) QUANTISEQ algorithms. (K) DC exhibited increased infiltration in the high‐risk group, which was notably pronounced in the xCell and (L) TIMER algorithms. (M) The high‐risk group demonstrated decreased tumour purity. (N) The high‐risk group exhibited elevated scores for various signatures related to immune function.

According to the above results, the PRS showed a close association with immune‐related functions and pathways. To avoid algorithm bias, seven immune algorithms, namely, CIBERSORT, QUANTISEQ, MCPcounter, xCell, ESTIMATE, EPIC and TIMER, were employed to comprehensively estimate the abundance of tumour‐infiltrating immune cells (TIICs) (Figure 5A). Notably, significant differences were observed in the tumour immune microenvironment (TIME) characteristics among distinct PRS score risk subgroups.

According to the results of MCPcounter, T cells, cytotoxic lymphocytes, CD8^+^ T cells and monocytic lineage were more abundant in the high‐risk group, while neutrophils were more abundant in the low‐risk group. EPIC analysis indicated a higher infiltration levels of CD8^+^ T cells and macrophages in the high‐risk group. xCell analysis revealed that CD8^+^ T cells, CD8^+^ naïve T cells, CD8^+^ Tcm, chondrocytes, dendritic cells (DC), aDC, macrophages M1, mast cells and regulatory T cells (Tregs) were more abundant in the high‐risk group. In contrast, epithelial cells, keratinocytes and sebocytes were more abundant in the low‐risk group. According to CIBERSORT, gamma delta T cells were more abundant in the high‐risk group, and B memory cells were more prevalent in the low‐risk group. QUANTISEQ analysis demonstrated more Tregs in the high‐risk group than in the low‐risk group, whereas monocytes and CD4^+^ T cells were more abundant in the low‐risk group. TIMER analysis indicated increased infiltration of CD8^+^ T cells and DC in the high‐risk group.

Furthermore, the high‐risk group exhibited significantly elevated scores, including the ImmuneScore (Figure 5B) and StromalScore (Figure 5C), as determined by the ESTIMATE algorithm. Additionally, the xCell algorithm revealed higher ImmuneScore (Figure 5D) in the high‐risk group. Notably, consistent results across various algorithms indicated a greater infiltration of CD8^+^ T cells, Tregs and DC in the high‐risk group. CD8^+^ T cells exhibited greater infiltration in the high‐risk group, as indicated by the MCPcounter (Figure 5E), EPIC (Figure 5F), xCell (Figure 5G) and TIMER algorithms (Figure 5H) and demonstrated increased infiltration levels based on the CIBERSORT (Figure S4A) and QUANTISEQ (Figure S4B) algorithms, although the difference was not statistically significant. Tregs infiltration level increased in the high‐risk group, particularly using the xCell (Figure 5I) and QUANTISEQ (Figure 5J) algorithms. DC exhibited increased infiltration levels in the high‐risk group, notably pronounced according to the xCell (Figure 5K) and TIMER (Figure 5L) algorithms. However, monocyte differences were not consistently observed between the two groups across different algorithms (Figure S4C‐F). The high‐risk group demonstrated increased enrichment of immune cells (Figure 5A–L) and decreased tumour purity (Figure 5M). In addition, this group exhibited elevated scores for various signatures related to immune function (Figure 5N), including chemokine receptor (CCR), checkpoint marker, cytolytic activity, inflammation promotion, HLA expression, MHC‐I expression, para‐inflammation and T cell co‐inhibition.

In brief, diverse immune algorithms revealed a higher abundance of specific immune cells and elevated immune function scores in the high‐risk group. Although the greater infiltration of immune cells in the high‐risk group, the overall immune status appears complex.

### 3.7 Immune cytokines and features in different PRS score risk subgroups

**FIGURE 6:**
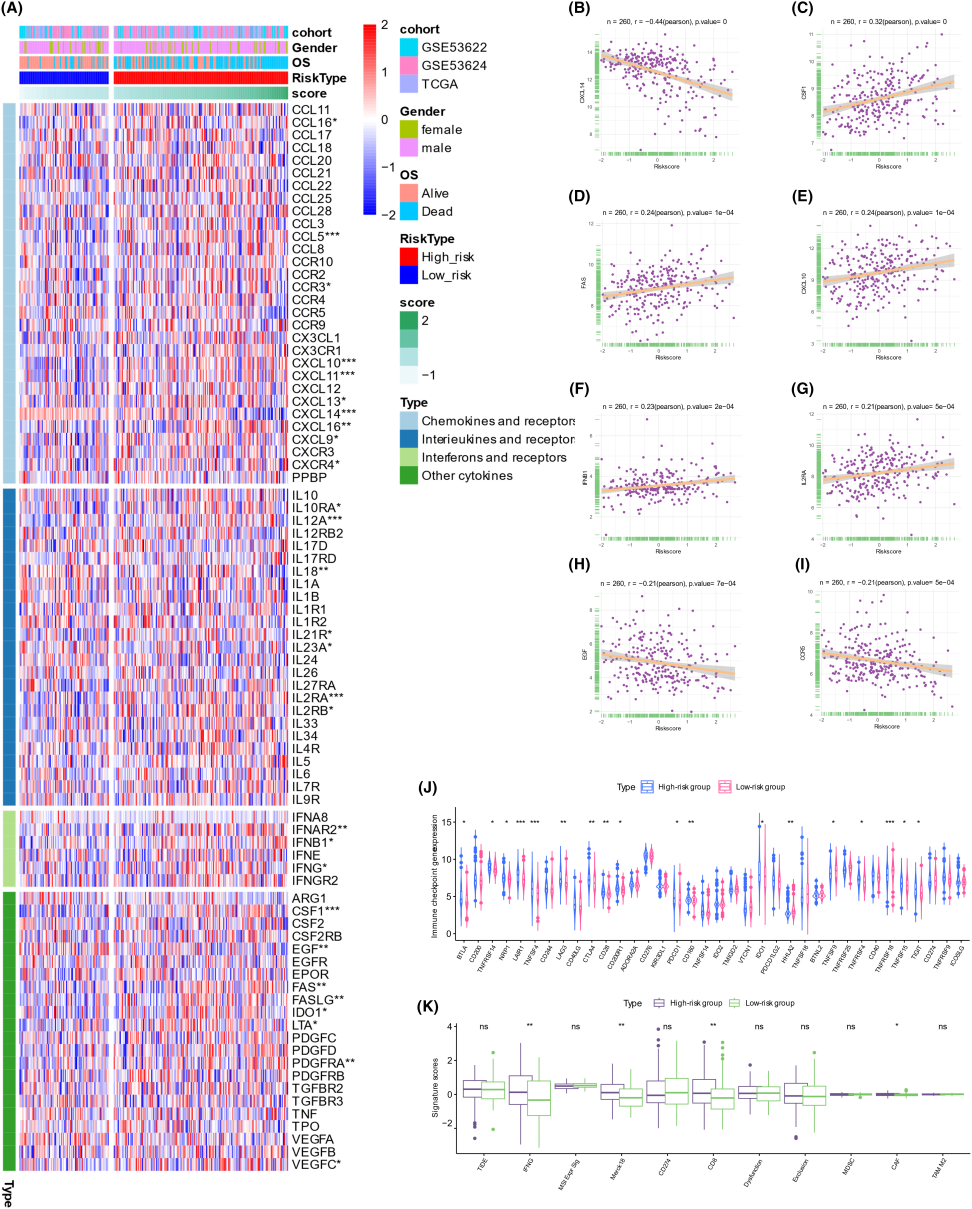
Immune cytokines and features in different PRS subgroups. (A) Cytokine and cytokine receptor levels were assessed in the high‐risk and low‐risk groups. (B) There were significant associations between CXCL14 (*r* = −0.44), (C) CSF1 (*r* = 0.32), (D) FAS (*r* = 0.24), (E) CXCL10 (*r* = 0.24), (F) IFNB1 (*r* = 0.23), (G) IL2RA (*r* = 0.21), (H) EGF (*r* = −0.21) and (I) CCR5 (*r* = −0.21) and the PRS score. (J) Fifteen immune checkpoint genes exhibited increased expression in the high‐risk group, while 3 genes were more pronounced in the low‐risk group. (K) IFNG, Merck18, CD8 and CAF showed higher levels in the high‐risk group.

Cytokine and cytokine receptors levels were assessed between the high‐ and low‐risk groups (Figure 6A–I). The findings revealed elevated levels of nine chemokines and CCRs, CCL16, CCL5, CCR3, CXCL10, CXCL11, CXCL13, CXCL16, CXCL9 and CXCR4; five interleukins and receptors: IL10RA, IL12A, IL21R, IL2RA and IL2RB; three interferons and receptors: IFNAR2, IFNB1 and IFNG; seven other cytokines: CSF1, FAS, FASLG, IDO1, LTA, PDGFRA and VEGFC, in the high‐risk group (Figure 6A). The levels of several cytokines, including CXCL14, IL18, IL23A and EGF, were lower in the high‐risk group (Figure 6A). There were significant associations between CXCL14 (*r* = −0.44), CSF1 (*r* = 0.32), FAS (*r* = 0.24), CXCL10 (*r* = 0.24), IFNB1 (*r* = 0.23), IL2RA (*r* = 0.21), EGF (*r* = −0.21) and CCR5 (*r* = −0.21) expression and the PRS score (Figure 6B–I).

Moreover, immune checkpoint genes, NRP1, LAIR1, BTLA, TNFRSF14, TNFSF4, CTLA4, CD28, LAG3, CD200R1, PDCD1, CD160, IDO1, TNFRSF4, TNFSF15 and TIGIT, were overexpressed in the high‐risk group, whereas HHLA2, TNFSF9 and TNFRSF18 were more pronounced in the low‐risk group (Figure 6J). Additionally, IFNG, Merck18, CD8 and CAF wereoverexpressed in the high‐risk group (Figure 6K), whereas other related markers showed no significant differences between groups (Figure 6K, Figure S4G, H). In addition, drug sensitivity prediction was conducted to predict the responsiveness to conventional chemotherapeutic drugs for the treatment of ESCC; however, no significant differences were observed (Figure S4I‐L).

In conclusion, the high‐risk group exhibited elevated gene expression levels of cytokines, cytokines receptors, immune checkpoint genes and improved immunotherapy response scores, suggesting a greater possibility of benefiting from immunotherapy among patients in the high‐risk group.

### 3.8 Gene expression distribution of PRS on distinct cell types on single‐cell level

**FIGURE 7:**
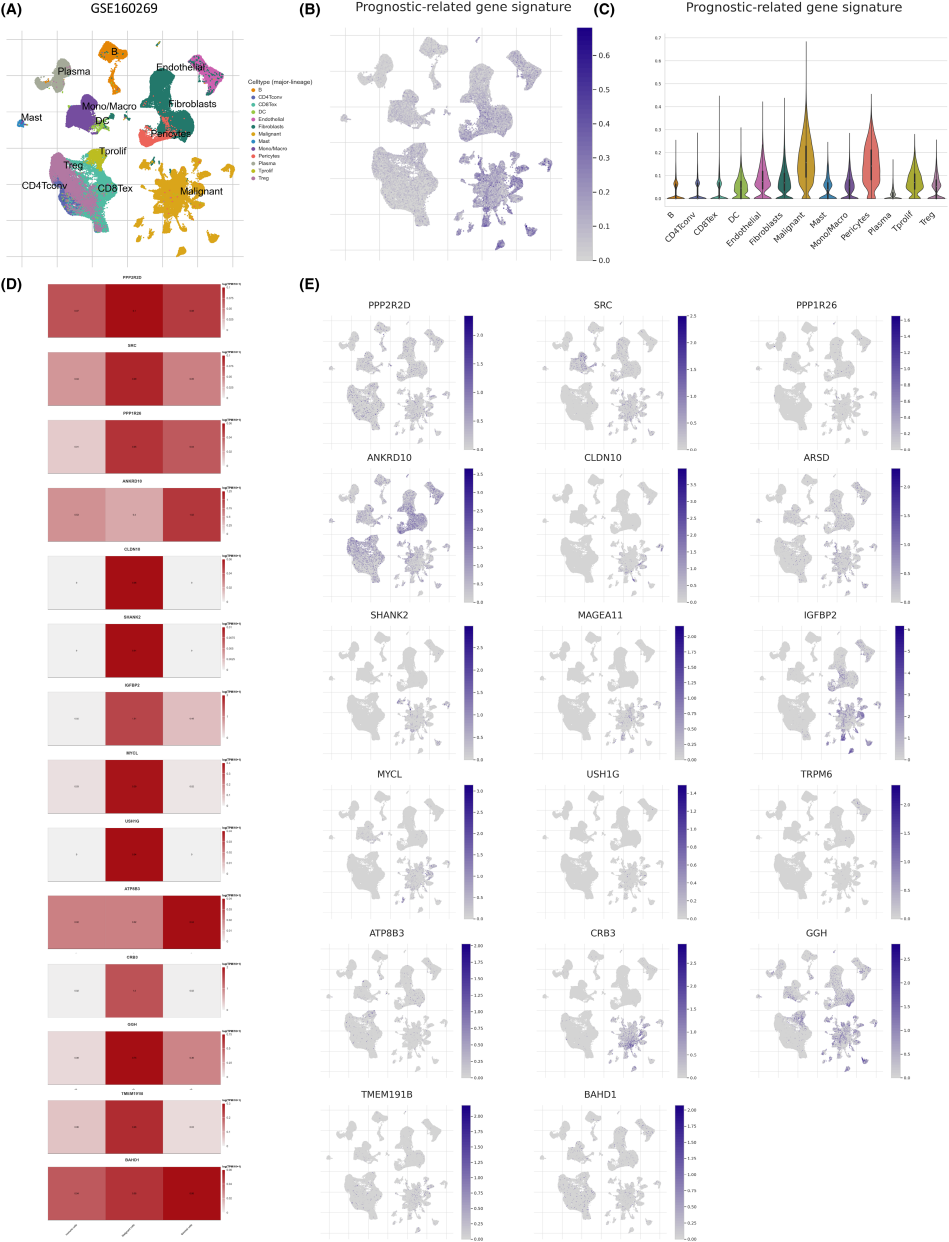
Gene expression distribution of PRS on distinct cell types on single cell level. In the GSE16029 dataset, (A) 13 major lineage cell populations were identified. (B) The UMAP plot displayed and (C) the grid violin plot detailed the average expression distribution of the 17‐gene on each type of cells. (D) The gene proportion were shown individually in three cell subtypes visualized by heatmaps, including immune, malignant and stromal cells. (E) 17‐gene expression were explored in 13 major lineage cell populations visualized by UMAP diagrams.

Our results indicated that the RPS was closely related to the TIME. Whether these 17 genes are expressed in tumour cells or immune cells remains to be determined. The expression distribution of the 17 genes was explored across malignant tumour cells and various TIICs using a single‐cell ESCC dataset (GSE16029).^40^, ^41^ Thirteen major lineage cell populations were identified, such as B cells, T cells and myeloid cells from the CD45^+^ cells and epithelial cells, endothelial cells, fibroblasts, pericytes and fibroblastic reticular cells from the CD45^−^ cells (Figure 7A). In the GSE16029 dataset, the UMAP plot was displayed (Figure 7B) and the grid violin plot showed (Figure 7C) the average expression distribution of the 17 genes in each type of cells. These results showed that the PRS was mainly expressed in malignant, pericytes and tprolif cells, with the highest expression in malignant cells (Figure 7B,C). Grid violin plots were used to display the expression level of 17 genes in detail (Figure S4M). Gene expression was shown individually in the three cell subtypes visualized by heatmaps (Figure 7D), including immune, malignant and stromal cells. Among them, PPP2R2D, SRC, PPP1R26, CLDN10, SHANK2, IGFBP2, MYCL, USH1G, CRB3, GGH and TMEM191B showed the highest expression in malignant cells, whereas ANKRD10, ATP8B3 and BAHD1 showed the highest expression in stromal cells.

Furthermore, the expression of 17 genes was explored in 13 major cell lineage and visualized by UMAP diagrams (Figure 7E). PPP2R2D, SRC, PPP1R26, CLDN10, ARSD, SHANK2, MAGEA11, IGFBP2, MYCL, USH1G, TRPM6, CRB3, GGH and TMEM191B were highly expressed in malignant cells. ANKRD10 was highly expressed in pericytes, fibroblasts, Tregs and endothelial cells. ATP8B3 was highly expressed in tprofil. In addition to being highly expressed in malignant cells, GGH was also highly expressed in tprofil, pericytes, endothelial cells and fibroblasts, while BAHD1 was uniformly distributed across most cell types.

These findings revealed that the PRS was closely related to the TIME at the single‐cell level and may potentially play a critical role in tumour immune regulation.

### 3.9 Investigation of the molecular features of distinct PRS score risk subgroups

To further investigate the molecular differences between the high‐ and low‐risk groups, gene transcription levels, CNVs and somatic mutations were assessed. The expression of the 17‐genes differed between high‐ and low‐ PRS score risk subgroups. The expression levels of ten genes decreased in the high‐risk group, such as SHANK2, IGFBP2, TMEM191B and TPRM6. In contrast, the expression levels of five genes increased, such as USH1G, PPP1R26 and CLDN10 (Figure S5A). Notably, the frequency of copy number amplifications or deletions varied significantly between these genes (Figure S5B). For instance, the frequency of CNV gain (%) of SHANK2 was considerably higher than that of CNV loss (%). In contrast, the CNV gain frequency (%) of ATP8B3 was considerably lower than its CNV loss frequency (%), which corresponded to alterations in gene transcription. Waterfall plots revealed high‐frequency mutations in different PRS subgroups. Genomic mutations were found in 92.98% of the high‐risk patients, with TP53 (79%), TTN (33%) and FLG (16%) as the top 3 altered genes (Figure S5C). In the low‐risk group, 95.83% patients exhibited mutations, with the top 3 genes exhibiting alteration in TP53 (71%), PIK3CA (29%) and CSMD3 (25%) (Figure S5D). Notably, TP53 emerged as the gene with the most frequently mutation rate in both the high‐ and low‐risk groups, with the high‐risk group showing an 8% higher mutation frequency than the low‐risk group. This genomic heterogeneity provides insight into the intricate differences underlying PRS score risk subgroups.

## 4 DISCUSSION

ESCC is a highly lethal disease with significant variable survival rates.^42^ Previous studies have developed various models to predict the OS for patients with ESCC.^6^, ^7^, ^8^, ^9^, ^10^, ^11^, ^12^, ^13^, ^17^, ^18^, ^19^, ^20^, ^21^, ^22^, ^23^, ^24^ However, challenges have been encountered in the development of predictive models for ESCC. This study provided a highly reliable predictive model for ESCC that showed notable differences compared with previous studies. First, the datasets included in the previous prognostic model studies were insufficient.^17^, ^18^, ^19^, ^20^, ^21^, ^22^ In this study, all available ESCC datasets were subjected to training or validation cohorts. Meanwhile, four metrics were estimated to avoid problems caused by overfitting. Second, the variables in previous studies were clinical parameters, pathological indicators and known gene signatures, such as ferroptosis, programmed necrosis and autophagy related genes. In this study, the 17 genes from the PRS model were selected based on univariate Cox regression analysis, which was more targeted for prognostic models. Third, no systematic and unbiased evaluation has been performed to assess the performance of the previous ESCC prognostic models. To fill this void, nine predictive model for ESCC and other two cancer types were utilized. Fourth, this study represents the first comprehensive evaluation of the performance of 10 ML algorithms to mitigate the impact of algorithms selection on analyses in constructing predictive models for ESCC, which generated 99 ML models.

Notheless, there are still several limitaions in this study. First, substantial amounts of high‐quality and comprehensive data are required for effective training to ensure the stability and accuracy of model development.^43^, ^44^, ^45^ Although all available public ESCC datasets were included, only the three cohorts were accessible, limiting the patient size to 260. This adversely affected the development of predictive models. Second, this model contained an expansive gene set of 17 genes, which increased the complexity of clincal translation. Future studies should focus on reducing the number of genes in the model while maintaining predictive performance. Third, although the findings were validated in external cohorts and two cohorts of other cancer types, experimental studies remain warranted to confirm the underlying mechanisms. Fourth, the clinical value of PRS should be more extensively confirmed in larger, multicenter and even multicancer cohorts to expand its application value. Fifth, the multi‐omics sequencing has provided richer data for constructing more robust prognostic models by ML.^27^, ^29^ Among them, more and more miRNAs have been identified, and there is increasing evidences that they may affect gene expression and disease progression.^46^, ^47^, ^48^, ^49^, ^50^ Integrating multiple dimensions of data, such as gene mutations,^14^ non‐coding RNA,^7^, ^28^, ^46^, ^47^, ^48^, ^49^, ^50^ and epigenomic,^15^, ^16^ will enhance the accuracy of model predictions. In the future, we intend to develop and upgrade prognostic models for ESCC using multi‐omics data.

Immunotherapy, primarily involving immune checkpoint inhibitors, is a rapidly developing strategy for tumour treatment in recent years^51^, ^52^ Nonetheless, there are still certain limitations in the current clinical efficacy of immunotherapy for ESCC. Predictive models based on ML and deep learning have been implemented to predict the efficacy of immunotherapy.^53^ This study revealed a close association between the PRS predictive model and pathways/functions related to immunity. Moreover, scRNA‐seq analysis revealed that the 17 genes of PRS were closely related to various immune cells in ESCC. Nevertheless, the absence of immunotherapy‐related data in patients with ESCC restricts a comprehensive assessment of the impact of the PRS model in predicting immunotherapy outcomes. With more datasets on immunotherapy for ESCC, we anticipate that the PRS predictive model will prove valuable in predicting the efficacy of immunotherapy for ESCC.

## 5 CONCLUSIONS

The 17‐gene PRS predictive model derived by RSF algorithm focused on enhancing the efficacy and precision of the predictive model and outperformed TNM systems and other previous predictive models, making it a robust model for OS prediction in ESCC. This model will contribute to the personalized treatment approaches and facilitate the prediction of immunotherapy efficacy in patients with ESCC.

## AUTHOR CONTRIBUTIONS

**Hongdian Zhang:** Conceptualization (lead); funding acquisition (lead); writing – review and editing (equal). **Peng Tang:** Data curation (lead); funding acquisition (supporting); writing – original draft (lead). **Baihui Li:** Conceptualization (supporting); funding acquisition (supporting); visualization (equal); writing – original draft (equal); writing – review and editing (equal). **Zijing Zhou:** Data curation (supporting); visualization (equal); writing – original draft (supporting). **Haitong Wang:** Investigation (equal). **Mingquan Ma:** Formal analysis (supporting). **Lei Gong:** Funding acquisition (supporting); writing – review and editing (supporting). **Yufeng Qiao:** Formal analysis (supporting). **Peng Ren:** Writing – review and editing (supporting).

## FUNDING INFORMATION

This work was supported by grants from the National Natural Science Foundation of China (grant No.82002551 and No.82403866), Bethune Charitable Foundation‐Excelsior Surgical Fund (CESS2021TB01), Tianjin Key Medical Specialty Construction Project (TJYXZDXK‐010A), Bethune Charity Foundation (HZB‐20190528‐11 and HZB‐20190528‐18), Clinical Trial Project of Tianjin Medical University (2017kylc006) and Tianjin Key Medical Discipline (Specialty) Construction Project (TJYXZDXK‐010A).

## CONFLICT OF INTEREST STATEMENT

The authors declare no conflicts of interest.

## CONSENT

All authors have approved this manuscript for publication.

## Supporting information


Data S1.

## Data Availability

R codes implemented in this study are available on GitHub (https://github.com/zhdiantjzl/ESCC_predict). Data used during this study are available from the corresponding author on reasonable request.
