# Supplementary material for: Integrated machine learning developed a prognosis‐related gene signature to predict prognosis in oesophageal squamous cell carcinoma
Source: J Cell Mol Med. 2024 Nov 13;28(21):e70171. doi: 10.1111/jcmm.70171 (PMC11558266; doi:10.1111/jcmm.70171)

**Integrated machine learning developed a prognosis-related gene signature to predict prognosis in esophageal squamous cell carcinoma**

Peng Tang ^1#^, Baihui Li ^1#✉^, Zijing Zhou ^2#^, Haitong Wang ^1^, Mingquan Ma ^1^, Lei Gong ^1^, Yufeng Qiao ^1^, Peng Ren ^1^, Hongdian Zhang ^1^^✉^

^1^ Tianjin Medical University Cancer Institute & Hospital, National Clinical Research Center for Cancer, Tianjin's Clinical Research Center for Cancer, Tianjin Key Laboratory of Digestive Cancer, Department of Esophageal Cancer, Tianjin, China.

^2^ Tianjin Medical University Cancer Institute & Hospital, National Clinical Research Center for Cancer, Tianjin's Clinical Research Center for Cancer, Key Laboratory of Cancer Prevention and Therapy, Department of Radiation Oncology, Tianjin, China.

^#^ These authors contributed equally to this work as co-first authors

^✉^ These authors contributed equally to this work as co-corresponding authors

Peng Tang tangpeng@tjmuch.com

Baihui Li libaihui@tjmuch.com

Zijing Zhou zhouzijing0425@163.com

Haitong Wang medicalwht@126.com

Mingquan Ma vamq2000@126.com

Lei Gong gonglei@tjmuch.com

Yufeng Qiao qyf0117@sina.com

Peng Ren renpeng2003@hotmail.com

Hongdian Zhang zhdiantjzl@tmu.edu.cn

**Running title:** Machine learning to predict prognosis in ESCC

**Keywords:** Esophageal squamous cell carcinoma, Machine-learning algorithm, Random survival forest, Tumor-infiltrating immune cells, Predictive model

****Corresponding author:***

**Hongdian Zhang Ph.D.**

Department of Esophageal Cancer, Tianjin Medical University Cancer Institute & Hospital, Huanhuxi Road, Tiyuanbei, Hexi District, Tianjin, P. R. China, 300060

Tel: 086-022-23340123

E-mail: zhdiantjzl@tmu.edu.cn

**Baihui Li Ph.D.**

Department of Esophageal Cancer, Tianjin Medical University Cancer Institute & Hospital, Huanhuxi Road, Tiyuanbei, Hexi District, Tianjin, P. R. China, 300060

Tel: 086-022-23340123

E-mail: libaihui@tjmuch.com

**Supplementary files:**

Number of figures: 5

**Supplementary materials and methods**

1. **The process of PRS predictive model by ML**

The RSF model was performed using the “randomForestSRC” package and the “rfsrc” function, incorporating parameters “ntree”, which represents the number of trees, and parameters “nodesize”, which represents the minimum size of the terminal nodes. In this study, “ntree” was set to 1,000 and “nodesize” was set to 5. The stepwise Cox model was utilized the “survival” package. All possible combination of the direction parameter were evaluated, including “forward”, “backward”, and “both”. The Lasso, Ridge regression, and Enet models were applied via the “glmnet” package and the “cv.glmnet” function. The regularization parameter lambda was established using 10-fold cross-validation, while the tradeoff parameter alpha was set between 0 and 1 (interval = 0.1); if alpha equaled 1, Lasso was performed, while alpha equaled 0, Ridge regression was performed, and Enet was performed for other values of alpha. The GBM model was implemented with the “gbm” package and the “gbm” function with 10-fold cross-validation. The survival-SVM model was analyzed using the “survivalsvm” package and the “survivalsvm” function, which employed support vector analysis on datasets with outcomes. The SuperPC model was utilized with “superpc” package and “superpc.cv” function, also incorporating 10-fold cross-validation. Lastly, the plsRcox model was performed via the “plsRcox” package and “cv.plsRcox” function, while the CoxBoost model was performed using the “CoxBoost” package and the “CoxBoost” function applying 10-fold cross-validation.

**Supplementary figure legend：**

**Figure S1 Data preprocessing and GO and KEGG enrichment analysis.**

**(A)** PCA plots before and **(B)** after the data were merged. **(C)** GO term annotation and **(D)** KEGG pathway enrichment analyses were conducted on the 17 genes, revealing the primary involvement of the genes in cell junctions.

**Figure S2 Stratified survival analysis of the RPS predictive model.**

**(A)** Patients with stage I-II, **(B)** stage III-IV, **(C)** G1-G2, **(D)** G3, **(E)** age < 65, **(F)** age ≥ 65, **(G)** female sex, or **(H)** male sex in the high-risk groups had a significantly shorter OS than those in the low-risk group in the Merge dataset. **(I, J)** No significant differences were observed among patients in different cohorts, or **(K, L)** between genders.

**Figure S3 Potential molecular mechanism related to the PRS according to GO and KEGG analyses.**

**(A)** The top 50 genes with a positive correlation and **(B)** negative correlation are visually represented using heatmaps. **(C)** Genes positively associated with the PRS (top 500 genes) were predominantly involved in cilium functions and antigen processing and presentation in terms of BP. **(D)** In the CC category, the term “membrane” was the primary representation. **(E)** Among the MF categories, peptide antigen binding and MHC protein complex binding were enriched. **(F)** These genes were shown to be related mainly to infection, antigen processing and presentation, and CAMs by KEGG analysis.

**Figure S4 Supplementation of the immune microenvironment and characteristic analysis of different PRS subgroups.**

**(A)** CD8^+^ T cells exhibited increased infiltration according to CIBERSORT and **(B)** QUANTISEQ, although the difference was not statistically significant. **(C-F)** Differences in monocytes levels were not consistently observed between the two groups across the different algorithms. **(G)** There are no significant differences in MSI or **(H)** TMB between the groups. **(I-L)** Drug sensitivity prediction revealed no response to conventional chemotherapy drugs in the treatment of ESCC. **(M)** Grid violin plots were used to display the expression level of 17-genes in details in scRNA-seq dataset.

**Figure S5 Investigation of molecular features in distinct PRS subgroups**

**(A)** Ten genes, including SHANK2, IGFBP2, TMEM191B, and TPRM6, were downregulated in the high-risk group; five genes, including USH1G, PPP1R26, and CLDN10, were upregulated. **(B)** The frequency of copy number amplifications or deletions varied significantly among these genes. **(C)** Genomic mutations were found in 92.98% of the high-risk group, with TP53 (79%), TTN (33%), and FLG (16%) emerging as the top 3 altered genes. **(D)** The low-risk group exhibited mutations in 95.83% of the patients, with the top 3 genes exhibiting alterations in TP53 (71%), PIK3CA (29%), and CSMD3 (25%).

**Figure S1 Data pre-processing and GO and KEGG enrichment analysis.**


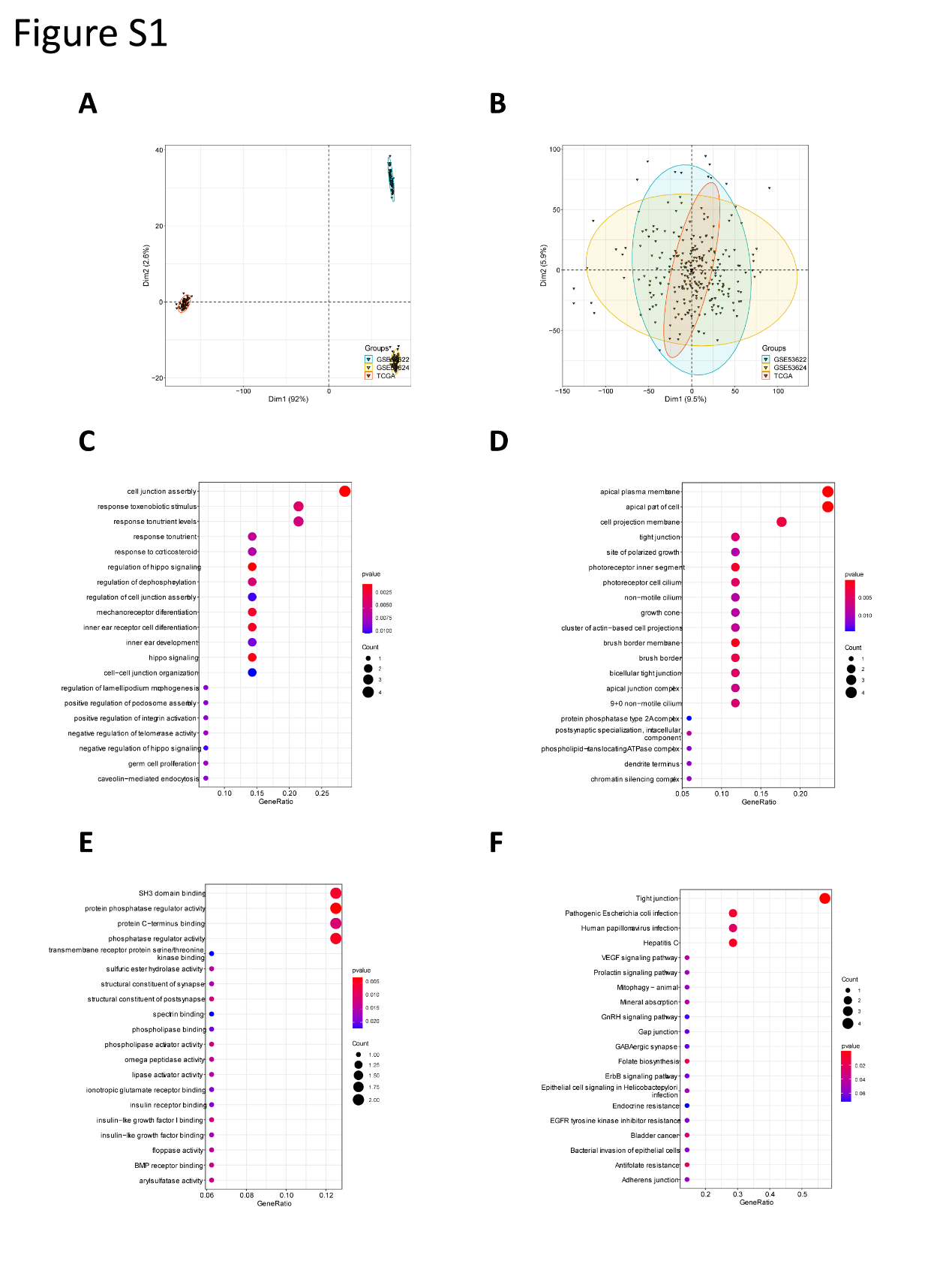


**Figure S2 Stratified survival and univariate Cox regression analysis of RPS predictive model.**

**
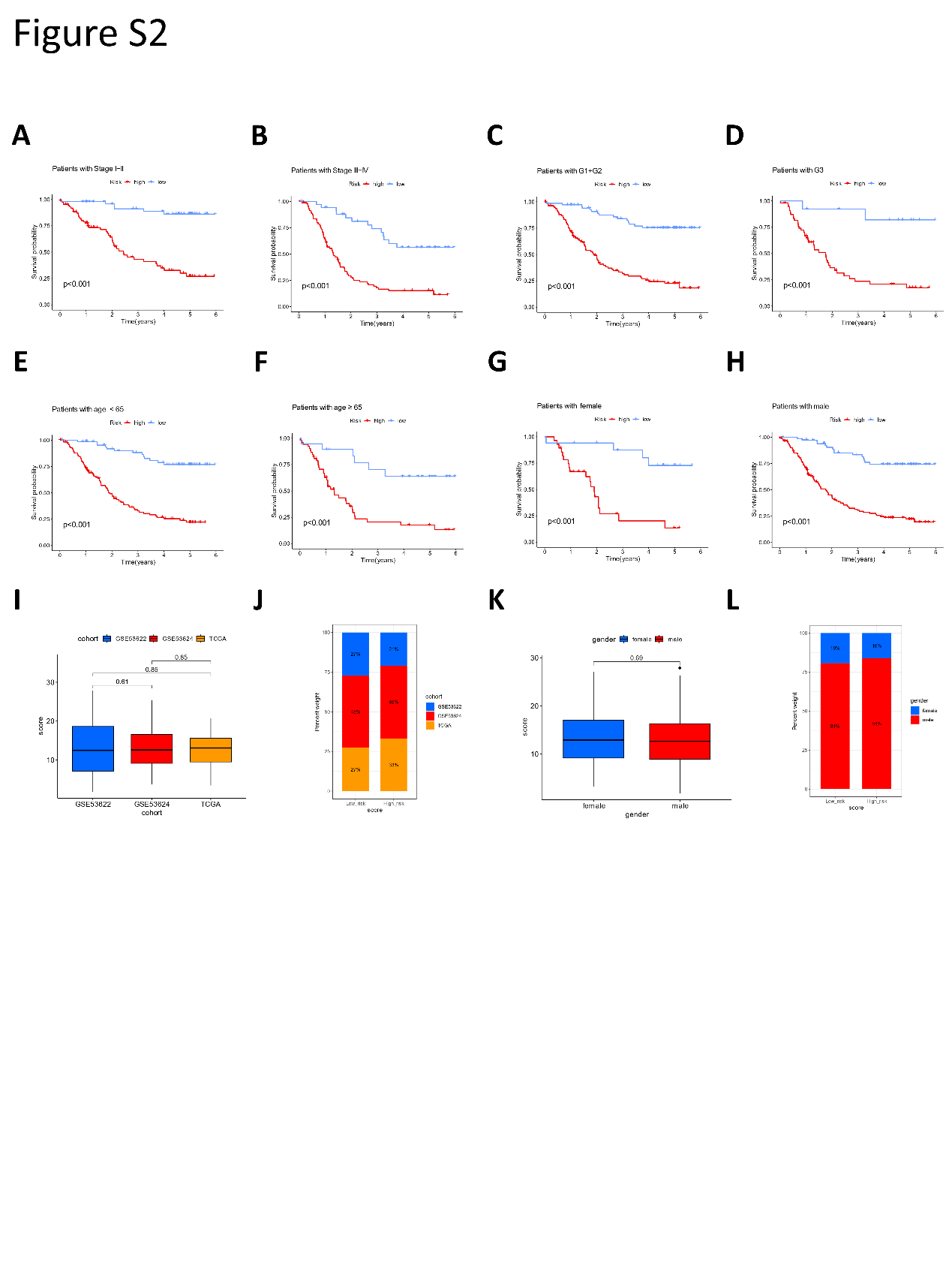
**

**Figure S3 Potential molecular mechanism related to the PRS by GO and KEGG analysis.**


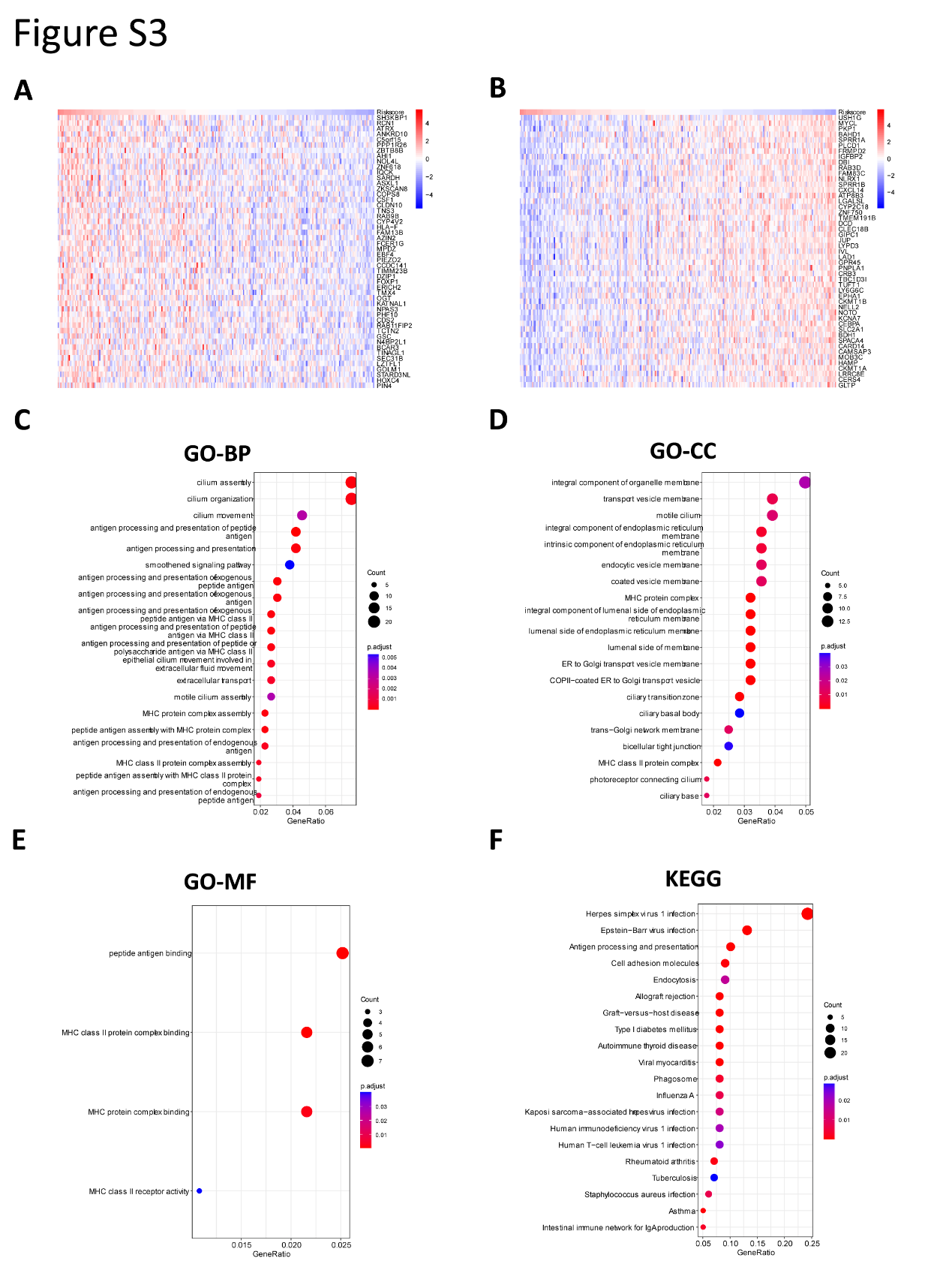


**Figure S4 Supplement for immune microenvironment and characteristics analysis in different PRS subgroups.**

**
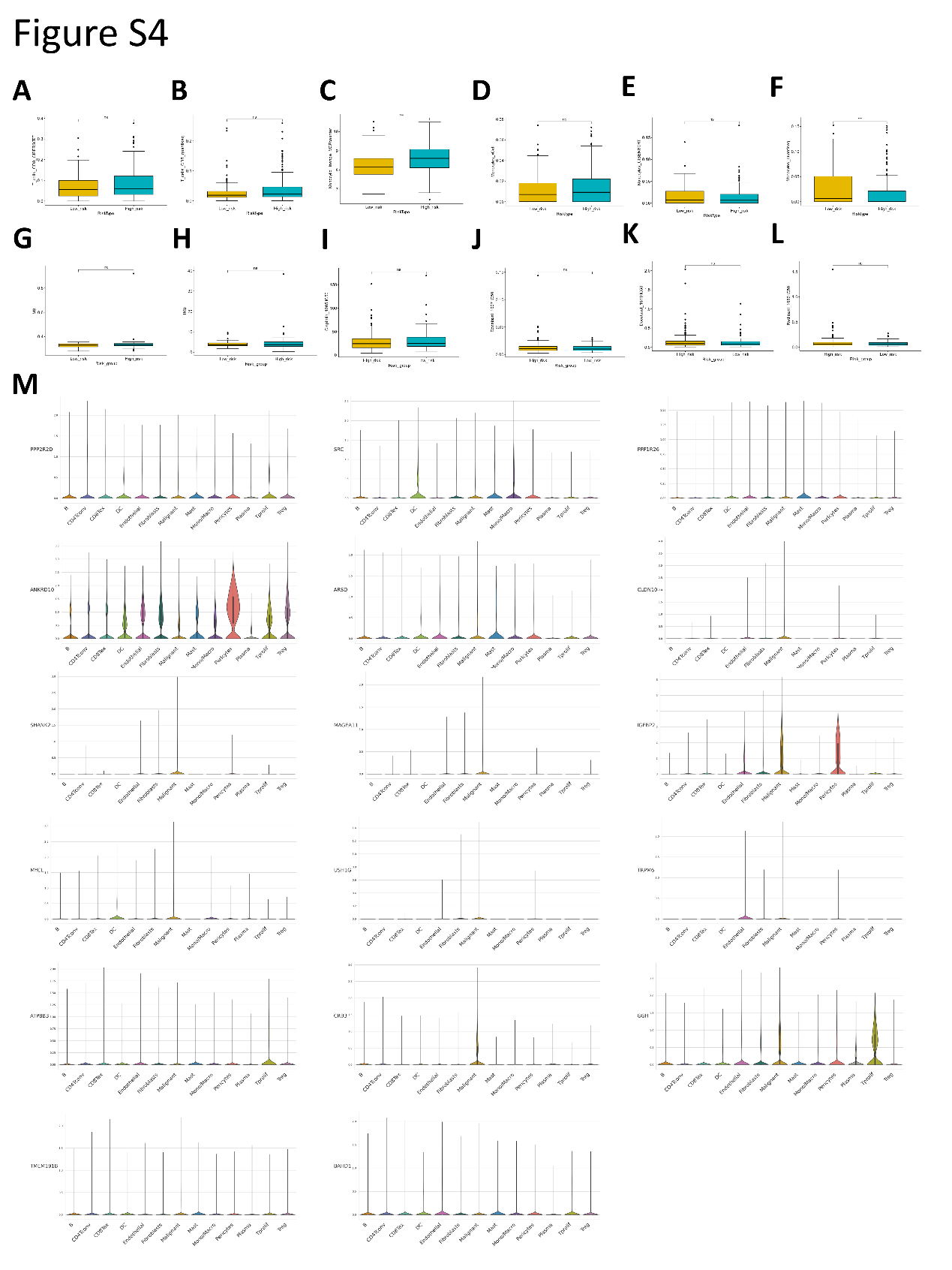
**

**Figure S5 Supplement to TMIT based on Siglec-15 and CD8^+^ T cells.**


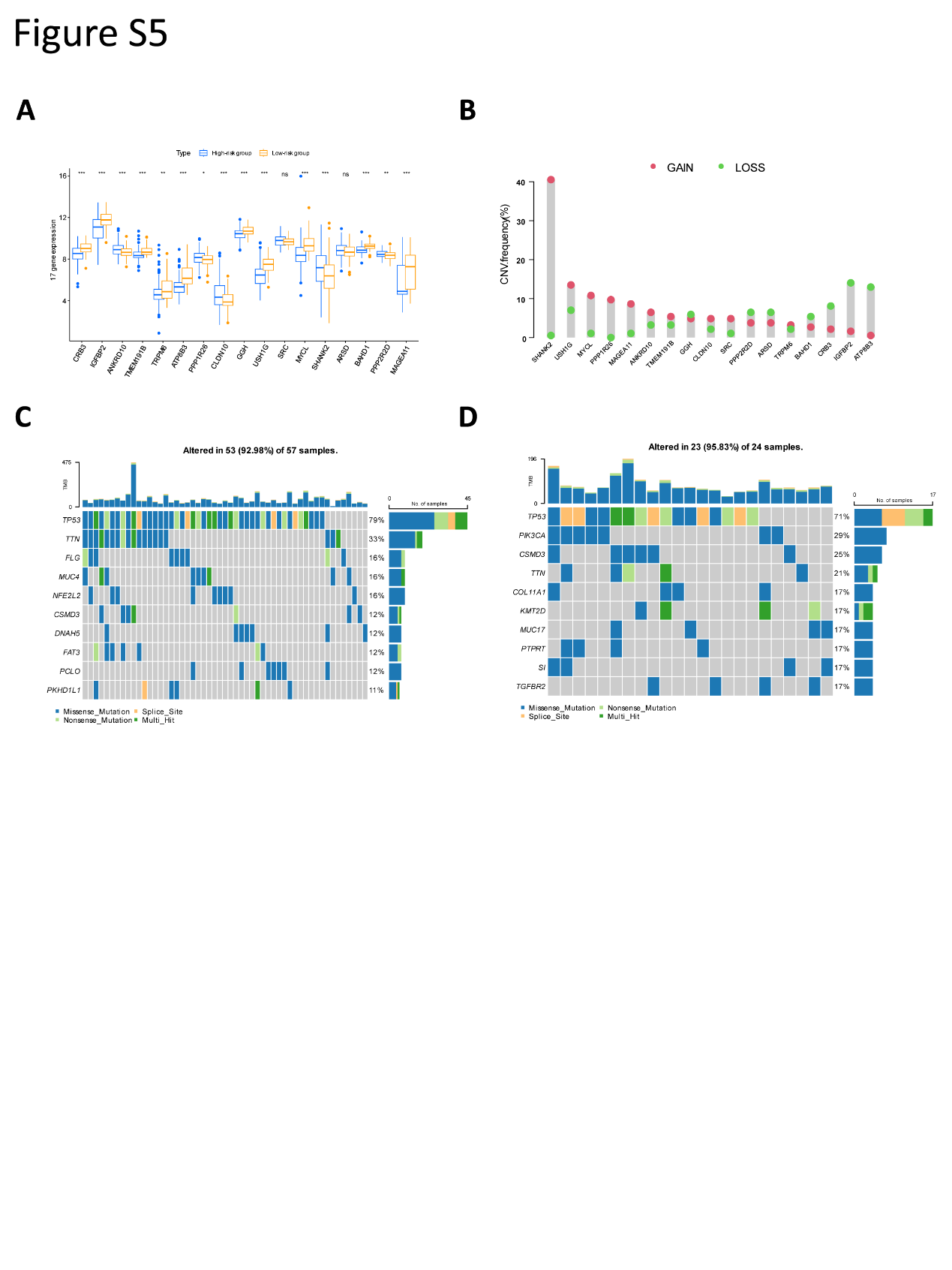

Supplement: Supplementary file 1 — Data S1. [file JCMM-28-e70171-s001.docx]
